# Prospective Study of Ultrasound-Guided Regional Anesthesia Blocks for Upper Extremity Procedures in a Community Emergency Medicine Hospital

**DOI:** 10.7759/cureus.117260

**Published:** 2026-09-30

**Authors:** Tristan Lilley, Natalie Siegfried, Ahmed Awab

**Affiliations:** 1 Emergency Medicine, Norman Regional Health System, Norman, USA; 2 Critical Care, Norman Regional Health System, Norman, USA

**Keywords:** emergency medicine, point-of-care ultrasound, procedural sedation, regional anesthesia, ultrasound

## Abstract

Painful upper extremity injuries frequently require procedures for which procedural sedation may be resource-intensive. This prospective study evaluated the educational impact and clinical feasibility of implementing a structured ultrasound-guided regional anesthesia curriculum as an alternative to procedural sedation for selected upper extremity procedures within a community emergency medicine residency.

From January through December 2025, attending emergency physicians participated in a two-hour didactic session and hands-on workshop followed by longitudinal on-shift mentorship. Physician knowledge was assessed using a locally developed pre- and post-intervention cognitive assessment. Seventeen physicians completed the curriculum. Mean assessment scores increased from 8.18±3.05 to 18.41±1.58, with a mean improvement of 10.24 points (95% confidence interval (CI), 9.02-11.45; p<0.001).

During clinical implementation, 30 ultrasound-guided brachial plexus blocks were performed. The intended procedure was completed without procedural sedation in 28 (93.3%) cases. In two (6.7%) cases, procedural sedation was subsequently required to complete the planned reduction. No immediate procedure-related complications were observed.

A structured ultrasound-guided regional anesthesia curriculum was associated with improved physician knowledge and successful clinical implementation of brachial plexus blocks. These findings support the feasibility of incorporating ultrasound-guided regional anesthesia as an additional analgesic strategy for selected upper extremity procedures in a community emergency medicine setting.

## Introduction

Painful upper extremity injuries, including fractures and joint dislocations, frequently necessitate prompt bedside analgesia and procedural intervention. While procedural sedation and analgesia (PSA) remains a standard and effective option, it requires intensive resource allocation, including dedicated nursing personnel, continuous cardiopulmonary monitoring, and prolonged recovery times [1,2]. Furthermore, PSA carries inherent cardiopulmonary risks and may be contraindicated in patients with complex comorbidities or non-fasting status [2].

Ultrasound-guided brachial plexus blocks, particularly supraclavicular and interscalene approaches, provide targeted, opioid-sparing analgesia that can mitigate the need for systemic sedation [3,4]. Despite established clinical efficacy and endorsement by emergency ultrasound guidelines [1], widespread adoption in community emergency departments is frequently hindered by limited educational infrastructure compared to academic centers with dedicated ultrasound fellowships. This study aimed to evaluate the implementation of a structured curriculum designed to transition physicians from didactic instruction to independent clinical competency, expanding the analgesic options available for painful upper extremity procedures.

## Materials and methods

Study design

This prospective educational and clinical implementation study was conducted from January 1, 2025, to December 31, 2025, at the Norman Regional Hospital Emergency Medicine Residency. The study was reviewed by the Norman Regional Health System Institutional Review Board (IRB), the institutional body responsible for ethical review of human-subject research, and was determined to meet criteria for exempt review (IRB Study #24-014). Formal exempt approval was granted on September 30, 2024. No ethical concerns or protocol-related ethical issues were identified during the study period.

The study consisted of an educational component and a subsequent clinical implementation component. The educational component evaluated physician cognitive knowledge before and after the implementation of a structured ultrasound-guided regional anesthesia curriculum. Following the educational intervention, clinical outcomes were prospectively collected during the implementation of ultrasound-guided regional anesthesia into emergency department practice. The clinical component was not designed as a controlled pre- and post-intervention comparison. Accordingly, pre-intervention patient-level clinical outcomes, including baseline rates of procedural sedation for comparable upper extremity procedures, were not prospectively collected. The pre- and post-intervention comparison was therefore limited to physician cognitive assessment scores.

The second component prospectively evaluated the clinical implementation of ultrasound-guided brachial plexus blocks following the introduction of the educational program. Clinical outcomes were collected for patients receiving blocks during the implementation period, including completion of the intended procedure without procedural sedation, subsequent requirement for procedural sedation, and observed immediate procedure-related complications. This clinical component was not designed as a controlled pre- and post-intervention comparison. Historical or prospectively collected pre-intervention patient-level data regarding the number of comparable upper extremity procedures, regional blocks performed, or procedural sedation utilization were not available. Accordingly, the study does not compare the utilization of procedural sedation before and after implementation and does not make conclusions regarding an overall reduction in the use of procedural sedation.

Inclusion and exclusion criteria

The educational intervention included attending emergency physicians within the department. For the clinical implementation phase, inclusion criteria comprised adult patients (≥18 years of age) presenting with acute, painful upper extremity injuries (fractures, dislocations, or complex lacerations) eligible for ultrasound-guided supraclavicular or interscalene brachial plexus blocks. The exclusion criteria included patient refusal, documented allergy to local anesthetics, active infection at the intended injection site, or underlying neurologic deficits in the affected extremity.

The number of patients evaluated for potential eligibility who ultimately did not undergo regional anesthesia was not systematically recorded; therefore, the clinical cohort represents patients who underwent ultrasound-guided brachial plexus block placement rather than all patients screened or assessed for potential inclusion.

Data collection

The curriculum was taught by Natalie Siegfried, DO, and supporting ultrasound faculty. The intervention consisted of a standardized two-hour didactic session and hands-on workshop. Educational impact was assessed using a locally developed 20-item cognitive assessment testing critical content covered in the educational intervention, including indications and contraindications for brachial plexus blocks, sonoanatomy, in-plane ultrasound-guided needle technique, local anesthetic pharmacology and dosing, procedural complications, and recognition and management of local anesthetic systemic toxicity (LAST). The same assessment instrument was administered immediately before and following the educational intervention.

Baseline physician performance on the pre-intervention cognitive assessment was used to characterize the educational gap targeted by the educational intervention. The pre-intervention mean assessment score was 8.18±3.05 out of a maximum possible score of 20, demonstrating an opportunity for structured education in the knowledge domains assessed by the instrument. The educational intervention was subsequently implemented to address this identified gap, followed by repeat administration of the same assessment instrument and prospective clinical implementation of the regional anesthesia program.

Each of the 20 items was equally weighted and scored using an all-or-none approach, with one point awarded for a completely correct response and zero points awarded for an incorrect or incomplete response, yielding a maximum possible score of 20 points. No partial credit was awarded. The assessment was locally developed for this educational intervention and was not subjected to formal psychometric validation. Accordingly, assessment scores were interpreted as measures of cognitive knowledge acquisition rather than as a demonstration of clinical or procedural competency. The complete assessment instrument is provided in the Appendices.

Longitudinal mentorship was operationalized via a “see one, do one, teach one” model during standard clinical shifts. Each participating physician was required to complete a minimum of one supervised block before being cleared for independent practice. Following this initial supervised procedure, physicians could perform additional blocks independently when clinically appropriate. Accordingly, the 30 blocks performed during the clinical implementation period included both the initial supervised procedures and subsequent blocks performed as part of routine clinical practice; physicians were not required to perform a predetermined number of additional blocks.

Statistical analysis

Data were analyzed using R version 4.2.2 (R Foundation for Statistical Computing, Vienna, Austria). Categorical variables were reported as frequencies and percentages (N (%)). Continuous variables (assessment scores) were reported as means with standard deviations (SD) and 95% confidence intervals (CI). Paired t-tests and Wilcoxon signed-rank tests were utilized to evaluate the pre- and post-intervention cognitive assessment scores. A p-value of <0.05 was considered statistically significant.

Clinical block protocol and medication safety

Ultrasound-guided brachial plexus blocks were performed using standardized medication-safety and monitoring practices. Local anesthetic selection and volume were individualized according to patient weight, clinical indication, and planned block. Physicians used either 5-10 mL of 0.5% ropivacaine diluted with 5-10 mL of sterile normal saline or a combination of 3 mL of 2% lidocaine and 5-7 mL of 0.5% ropivacaine diluted with 5-10 mL of sterile normal saline. Total local anesthetic doses were calculated according to patient weight and institutional maximum-dose guidance, with ropivacaine dosing limited to 2 mg/kg. Epinephrine was not routinely added to the local anesthetic solution.

Prior to block placement, a procedural time-out was performed and patients were placed on continuous cardiac and pulse oximetry monitoring. Patients remained monitored for a minimum of 30 minutes following block placement, with continued monitoring beyond this period when clinically indicated; in routine practice, post-procedure observation frequently extended to one hour or longer.

A hospital-approved protocol for recognition and management of LAST, including lipid emulsion therapy, was available throughout the study period. The LAST protocol was posted in common clinical areas and maintained with the procedural ultrasound equipment. Point-of-care procedural reference cards outlining block technique and medication-safety considerations were also maintained with the ultrasound equipment to facilitate standardized clinical practice.

## Results

Baseline educational assessment and gap identification

All 17 (100%) attending emergency physicians who participated in this curriculum completed the baseline cognitive assessment before the educational intervention. The mean pre-intervention assessment score was 8.18±3.05 out of a maximum possible score of 20, and the highest observed baseline score was 17 of 20. These baseline findings identified an opportunity for structured education in the knowledge domains assessed by the instrument, including indications and contraindications for brachial plexus blocks, sonoanatomy, ultrasound-guided needle technique, local anesthetic pharmacology and dosing, procedural complications, and recognition and management of local anesthetic systemic toxicity.

Educational intervention and post-intervention outcomes

All 17 (100%) participating physicians completed the educational intervention and post-intervention assessment. Following the standardized didactic session, hands-on workshop, and longitudinal clinical mentorship, mean assessment scores increased from 8.18±3.05 to 18.41±1.58. The mean improvement was 10.24 points (95% CI, 9.02-11.45; p < 0.001). The highest observed post-intervention score was 20 of 20, and 16 of 17 (94.1%) physicians improved their baseline score by at least 50%. Because the assessment instrument was locally developed and was not formally psychometrically validated, these findings were interpreted as an improvement in assessed cognitive knowledge rather than a demonstration of procedural competency. Educational assessment and overall clinical implementation outcomes are summarized in Table 1.

**Table 1:** Educational Assessment Outcomes and Clinical Implementation Statistics

Outcome	Pre-intervention	Post-intervention / Implementation
Physicians completing assessment, N (%)	17 (100%)	17 (100%)
Cognitive assessment score, mean±SD	8.18±3.05	18.41±1.58
Mean change (95% CI)	-	+10.24 (9.02-11.45), p<0.001
Physicians improving baseline score ≥50%, N (%)	-	16 (94.1%)
Clinical implementation cohort	Not collected	30 (100%)
Ultrasound-guided brachial plexus blocks performed	Not collected	30 (100%)
Intended procedure completed without procedural sedation	Not collected	28 (93.3%)
Procedural sedation subsequently required	Not collected	2 (6.7%)
Immediate procedure-related complications observed	Not collected	0 (0.0%)

Clinical implementation outcomes

Following implementation of the educational program, 30 ultrasound-guided brachial plexus blocks were performed in the prospective clinical cohort. Of these, 14 of 30 (46.7%) were supraclavicular blocks and 16 of 30 (53.3%) were interscalene blocks.

The intended upper extremity procedure was completed following regional anesthesia without procedural sedation in 28 of 30 (93.3%) cases. All 14 of 14 (100%) patients receiving supraclavicular blocks underwent completion of the intended procedure without subsequent procedural sedation. Among the 16 patients receiving interscalene blocks, 14 of 16 (87.5%) underwent completion of the intended procedure without procedural sedation, while two of 16 (12.5%) subsequently required procedural sedation to complete the planned reduction.

Both patients requiring subsequent procedural sedation were men and had received interscalene blocks. One patient received etomidate and the other received propofol for procedural sedation. No patients receiving supraclavicular blocks required subsequent procedural sedation. No immediate procedure-related complications, including local anesthetic systemic toxicity, pneumothorax, or vascular injury, were observed in the 30-patient clinical implementation cohort. Clinical implementation outcomes, including completion of the intended procedure without procedural sedation and subsequent procedural sedation requirements according to block type, are summarized in Table 2.

**Table 2:** Clinical Implementation and Procedural Sedation Outcomes by Brachial Plexus Block Type

Clinical Implementation Outcome	N (%)
Total brachial plexus blocks performed	30 (100%)
Supraclavicular blocks	14 (46.7%)
Interscalene blocks	16 (53.3%)
Intended procedure completed without procedural sedation - overall	28/30 (93.3%)
Intended procedure completed without procedural sedation - supraclavicular	14/14 (100%)
Intended procedure completed without procedural sedation - interscalene	14/16 (87.5%)
Subsequent procedural sedation required - overall	2/30 (6.7%)
Subsequent procedural sedation required - supraclavicular	0/14 (0.0%)
Subsequent procedural sedation required - interscalene	2/16 (12.5%)
Etomidate used for subsequent procedural sedation	1/30 (3.3%)*
Propofol used for subsequent procedural sedation	1/30 (3.3%)*
Immediate procedure-related complications observed	0/30 (0.0%)

Standardized pain scores and formal sensory and motor block assessments were not prospectively collected. Age and sex were not systematically recorded for the entire clinical implementation cohort; therefore, demographic characteristics are not reported beyond the available characteristics of the two patients who subsequently required procedural sedation. Accordingly, completion of the intended procedure without procedural sedation was treated as an implementation outcome and not as an independent measure of block efficacy or adequacy of analgesia.

There was no pre-intervention patient-level clinical cohort. The pre- and post-intervention phases applied to the physician educational assessment rather than to patient-level clinical outcomes. Pre-intervention patient-level data, including the number of patients undergoing comparable upper extremity procedures, the number receiving procedural sedation, and baseline utilization of ultrasound-guided brachial plexus blocks, were not prospectively collected. Therefore, patient-level procedural and sedation outcomes are reported only for the prospective clinical implementation cohort, and no pre- versus post-intervention comparison of procedural sedation utilization was performed.

## Discussion

This prospective implementation study demonstrates that a structured ultrasound-guided regional anesthesia curriculum can successfully integrate brachial plexus blocks into routine emergency department practice within a community emergency medicine residency. By leveraging a "see one, do one, teach one" on-shift mentorship model alongside targeted didactics, community programs can effectively implement advanced point-of-care ultrasound procedures. The 10.24-point mean improvement in assessment scores demonstrates substantial improvement in knowledge of the content provided by the educational intervention. These findings are consistent with improvement in the physician's cognitive knowledge of this regional anesthetic technique, which is necessary for the procedure's safe execution [3].

Following implementation of a standardized educational program, physicians demonstrated significant improvement in procedural knowledge, successfully incorporated ultrasound-guided regional anesthesia into clinical practice, and avoided procedural sedation in the majority of patients. Importantly, implementation occurred without any observed immediate procedure-related complications, supporting both the feasibility and early safety of this educational model.

Although procedural sedation remains an essential component of emergency medicine practice, it requires appropriate patient assessment, physiologic monitoring, and personnel prepared to recognize and manage sedation-related adverse events [2]. These requirements may increase resource utilization and recovery time compared with procedures performed under regional anesthesia. Furthermore, the selection and performance of procedural sedation require consideration of patient-specific factors, appropriate physiologic monitoring, and the availability of personnel capable of monitoring the patient and managing potential complications [2]. Ultrasound-guided regional anesthesia may provide an alternative approach for selected patients by providing targeted analgesia while potentially reducing exposure to systemic sedative medications [1,3,4]. Our findings suggest that implementation of a structured regional anesthesia program can successfully expand the analgesic options available to emergency physicians without compromising procedural success.

The clinical outcomes observed support the integration of ultrasound-guided regional anesthesia into standard community emergency practice. Reducing reliance on systemic sedation not only circumvents the cardiopulmonary risks highlighted in clinical policies but also actively decongests department workflow by eliminating prolonged post-sedation recovery [2,4]. Furthermore, the absence of LAST or pneumothorax in this cohort aligns with literature affirming the safety profile of real-time sonographic needle tracking [5].

Ultrasound-guided peripheral nerve blocks have increasingly been described as effective techniques for providing targeted procedural anesthesia for painful extremity conditions in the emergency department [3,4]. The clinical outcomes observed in our study further support the integration of ultrasound-guided regional anesthesia into emergency department practice. Overall procedural sedation was avoided in nearly all patients following successful block placement. Although our study was not designed to evaluate emergency department throughput, patient satisfaction, opioid utilization, or healthcare costs, reducing the need for procedural sedation has the potential to improve efficiency while minimizing exposure to systemic sedative medications. Future investigations should evaluate these patient-centered and operational outcomes to better define the broader impact of regional anesthesia implementation.

Overall, our experience demonstrates that implementation of a structured ultrasound-guided regional anesthesia curriculum is both feasible and clinically effective within a community emergency medicine program. By improving physician competency while expanding the availability of regional anesthesia, this educational model offers a practical strategy for reducing reliance on procedural sedation and enhancing analgesic care for patients undergoing painful upper extremity procedures.

This study has several limitations. It was conducted at a single community residency center with a small sample size of 17 (100%) attending physicians and a clinical convenience sample of 30 patients, limiting broad generalizability and statistical power for detecting rare adverse events. An additional limitation is the absence of pre-intervention patient-level clinical data. Baseline numbers of comparable upper extremity procedures, procedural sedation utilization, and ultrasound-guided brachial plexus block utilization were not prospectively collected before implementation. Consequently, although 28 (93.3%) procedures in the prospective implementation cohort were completed without procedural sedation, these findings cannot establish that implementation of the regional anesthesia program reduced overall procedural sedation utilization compared with prior practice. The pre- and post-intervention component of this study therefore applies to physician cognitive knowledge, whereas the patient-level clinical component should be interpreted as a prospective implementation cohort. Long-term knowledge retention, exact emergency department length-of-stay metrics, patient-reported pain scores, formal sensory and motor block assessments, and longitudinal patient follow-up were not evaluated during this initial implementation phase. Future studies should incorporate prospective baseline and post-implementation clinical cohorts to permit direct comparison of procedural sedation utilization and other patient-centered outcomes.

## Conclusions

A structured ultrasound-guided regional anesthesia curriculum combining focused didactic education, hands-on training, and longitudinal on-shift mentorship improved physician knowledge and aided in the successful integration of brachial plexus blocks into clinical practice within a community emergency medicine residency. The findings demonstrate the feasibility of implementing ultrasound-guided regional anesthesia as an additional analgesic strategy for appropriately selected patients undergoing painful upper extremity procedures and suggest that these techniques may provide an alternative to procedural sedation in selected cases.

This educational model may offer a practical framework for community emergency departments seeking to expand physician knowledge and clinical experience in ultrasound-guided regional anesthesia. However, given the single-center design and limited clinical sample, the absence of observed immediate adverse events should be interpreted descriptively and should not be considered evidence establishing procedural safety or a low complication rate. Larger multicenter studies with systematic follow-up are needed to evaluate the durability of physician competency, patient-centered outcomes, procedural complications, emergency department resource utilization, and the broader effect of regional anesthesia programs on procedural sedation use.

## Appendices

Locally developed regional anesthesia cognitive assessment

*Assessment Introduction* 

Welcome to the Interscalene and Supraclavicular block PRE training test.

These innovative blocks are designed to give the emergency physician additional tools in treating patients in the department. 

Much like the fascia iliaca block, the supraclavicular and the interscalene blocks are designed for pain control for the patient who has extensive arm wounds, dislocations, fractures, or other pathology involving the upper extremity. 

The results are private, will not be shared, and are being used to track improvement.

Assessment Questions 

1. True or False: This image depicts the anesthetic coverage of an interscalene block.

○ True

○ False 

2. True or False: This image depicts the anesthetic coverage of a supraclavicular block. 

○ True 

○ False 

3. What is reliably anesthetized by an interscalene block? 

○ Shoulder, Upper Arm and Lateral 2/3 Clavicle 

○ Hand, Lower Arm and Elbow 

○ Neck, Shoulder and Medial 2/3 Clavicle 

4. What is reliably anesthetized by a supraclavicular block? 

○ Shoulder, Upper Arm and Lateral 2/3 Clavicle 

○ Hand, Lower Arm and Elbow 

○ Neck, Shoulder and Medial 2/3 Clavicle 

5. Which of the following is an indication for a brachial plexus nerve block? Select all that apply. 

○ Closed Fracture Reduction 

○ Dislocation Reduction 

○ Wound Closure 

○ Wound Exploration & Irrigation 

○ Refractory Pain 

○ Unconsenting or Confused Patient 

○ Limb Ischemia 

○ Respiratory Insufficiency 

○ Compartment Syndrome 

○ Active Infection 

○ Local Anesthetic Allergy 

○ Coagulopathy 

6. Which of the following is a contraindication for a brachial plexus nerve block? Select all that apply. 

○ Closed Fracture Reduction 

○ Dislocation Reduction 

○ Extremity Wound Closure 

○ Wound Exploration and Irrigation 

○ Unconsenting or Confused Patient 

○ Limb Ischemia 

○ Respiratory Insufficiency 

○ Compartment Syndrome 

○ Active Infection 

○ Local Anesthetic Allergy 

○ Coagulopathy 

7. True or False: This image depicts an interscalene nerve block window.

○ True 

○ False 

8. True or False: This image depicts a supraclavicular nerve block window.

○ True 

○ False 

9. True or False: The easiest way to find the interscalene ultrasound window is by first finding and then scanning up from the supraclavicular ultrasound window.

○ True 

○ False 

10. True or False: Out-of-plane needle guidance is the most common technique for brachial plexus blocks. 

○ True 

○ False 

11. The needle path for the interscalene block traverses which structures? Where is the local anesthetic drop point?

Select one that applies for each. 

○ Skin > Subcutaneous Tissue > Cervical Fascia > Middle Scalene Muscle ○ Between the Cervical Fascia and the Upper Trunk; and between the Lower Trunk and the first Rib 

○ Skin > Subcutaneous Tissue > Cervical Fascia > Omohyoid muscle ○ Skin > Pleura > Lung 

○ Between the C5 and C6 nerve root fascicles 

12. The needle path for the supraclavicular block traverses which structures? Where is the local anesthetic drop point?

Select one that applies for each. 

○ Skin > Subcutaneous Tissue > Cervical Fascia > Middle Scalene Muscle ○ Between the Cervical Fascia and the Upper Trunk; and between the Lower Trunk and the first Rib 

○ Skin > Subcutaneous Tissue > Cervical Fascia > Omohyoid muscle ○ Skin > Pleura > Lung 

○ Between the C5 and C6 nerve root fascicles 

13. What are some direct complications of brachial plexus nerve blocks?

Select five that apply. 

○ Pneumothorax 

○ Pneumonia 

○ Block Failure 

○ Hematoma 

○ Permanent Paralysis 

○ Nerve Injury 

○ Myocardial Infarction 

○ Hemidiaphragmatic Paralysis & Respiratory Failure 

○ Stroke 

○ Local Anesthetic Systemic Toxicity 

○ Infection 

14. What is the maximum dose of Ropivacaine? What is the maximum dose of Lidocaine? What is the maximum dose of Bupivacaine? Which of the three is considered more cardiotoxic?

Select one that applies for each. 

○ Ropivacaine 2-3 mg/kg 

○ Ropivacaine is more cardiotoxic

○ Bupivacaine 4-5 mg/kg 

○ Bupivacaine is more cardiotoxic 

○ Bupivacaine 2-2.5 mg/kg 

○ Ropivacaine 4-5 mg/kg 

○ Lidocaine 7 mg/kg 

○ Lidocaine 4-5 mg/kg 

○ Lidocaine is more cardiotoxic 

15. What is Local Anesthetic Systemic Toxicity? What is the treatment for it?

Select one that applies for each. 

○ ACLS plus Intralipid 

○ Both neurologic and cardiovascular manifestations from systemically absorbed local anesthetic 

○ CNS toxicity manifesting as confusion, agitation, tinnitus, perioral paresthesia, tremors that may progress to seizure, coma, and respiratory arrest 

○ BLS plus observation 

○ CVS toxicity manifesting as tachycardia, hypertension followed by ventricular arrhythmias, hypotension and cardiac arrest 

16. What is the appropriate initial IV fat emulsion dose? Select one that applies. What modifications do you make to ACLS in the setting of LAST? Select all that apply.

○ 1.5 ml/kg bolus of 20% intralipid followed by 0.25 ml/kg/min infusion

○ Do NOT shock patient for unstable shockable arrhythmias 

○ Give reduced dose epinephrine (< 1 mcg/kg) every 2-5 min 

○ Do NOT give vasopressin or calcium channel blockers 

○ Avoid propofol 

○ 3.5 ml/kg bolus of 20% intralipid followed by 1 ml/kg/min infusion 

○ Give lidocaine for refractory ventricular arrhythmias 

○ Give benzodiazepines for seizure abortion 

17. What is the appropriate subsequent IV fat emulsion dose? What is the total maximum dose of intralipid?

Select one each that applies. 

○ 1.5 ml/kg re-bolus followed by increased rate to 0.5 ml/kg/min 

○ 3.5 ml/kg re-bolus followed by increased rate to 1 ml/kg/min 

○ Maximum dose of 30 ml/kg over 30 minutes 

○ Maximum dose of 10 ml/kg over 30 minutes 

18. The actions below are most important for avoiding complications of ultrasound-guided nerve blocks. True or False. 

○ True 

○ False 

19. Your patient has intact sensation immediately after performing a supraclavicular nerve block for radial fracture reduction.

Your next step is: 

○ Perform an interscalene block 

○ Inject 1 mg/kg of lidocaine into patient's IV 

○ Wait 15-20 minutes, then re-assess motor and sensation 

○ Do better next time

20. How long must a patient be monitored after brachial plexus nerve block placement? Which monitors should be used? What return precautions should you give?

Select one that applies for each. 

○ 30-45 minutes 

○ Pulse Oximetry, NIBP, EKG 

○ Neurologic, cardiovascular and respiratory-specific return precautions ○ 2 hours 

○ Abdominal return precautions 

○ Pulse Oximetry and Capnography alone
